# Forward Osmosis Application for the Removal of Emerging Contaminants from Municipal Wastewater: A Review

**DOI:** 10.3390/membranes13070655

**Published:** 2023-07-10

**Authors:** Mónica Salamanca, Mar Peña, Antonio Hernandez, Pedro Prádanos, Laura Palacio

**Affiliations:** 1 Institute of Sustainable Processes (ISP), Dr. Mergelina s/n, 47011 Valladolid, Spain; mar.penamiranda@uva.es (M.P.); antonio.hernandez@uva.es (A.H.); ppradanos@uva.es (P.P.); laura.palacio@uva.es (L.P.); 2 Department of Chemical Engineering and Environmental Technology, University of Valladolid, Dr. Mergelina s/n, 47011 Valladolid, Spain; 3 Department of Applied Physics, Faculty of Sciences, University of Valladolid, Paseo Belén 7, 47011 Valladolid, Spain

**Keywords:** municipal wastewater, contaminants, membranes, forward osmosis (FO)

## Abstract

Forward osmosis (FO) has attracted special attention in water and wastewater treatment due to its role in addressing the challenges of water scarcity and contamination. The presence of emerging contaminants in water sources raises concerns regarding their environmental and public health impacts. Conventional wastewater treatment methods cannot effectively remove these contaminants; thus, innovative approaches are required. FO membranes offer a promising solution for wastewater treatment and removal of the contaminants in wastewater. Several factors influence the performance of FO processes, including concentration polarization, membrane fouling, draw solute selection, and reverse salt flux. Therefore, understanding and optimizing these factors are crucial aspects for improving the efficiency and sustainability of the FO process. This review stresses the need for research to explore the potential and challenges of FO membranes to meet municipal wastewater treatment requirements, to optimize the process, to reduce energy consumption, and to promote scalability for potential industrial applications. In conclusion, FO shows promising performance for wastewater treatment, dealing with emerging pollutants and contributing to sustainable practices. By improving the FO process and addressing its challenges, we could contribute to improve the availability of water resources amid the global water scarcity concerns, as well as contribute to the circular economy.

## 1. Introduction

Water scarcity and contamination are considered serious problems of worldwide concern, in relation to both industrial requirements and population growth [1,2]. In addition to current water scarcity, it is estimated that water shortage could increase up to 60% by 2025 [3,4]. The sixth sustainable development goal of the 2030 agenda focuses on the availability and sustainable management of water and sanitation for all.

Therefore, an efficient management of water resources is necessary. In the prosecution of this aim, wastewater treatment plants (WWTPs) play a fundamental role. It should be noted that municipal WWTPs are designed to reduce pollution and to protect environmental quality and human health, in addition to obtaining benefits such as water, nutrients, and energy [5,6].

WWTPs are facilities that treat the wastewaters (WW) generated by an area or city; therefore, an increase in urban population directly influences WW discharges that must be controlled and treated so that they do not pose a risk to humans and the environment.

Increasing environmental constraints worldwide are creating the need to adapt conventional wastewater plants to more sustainable and robust treatment systems, employing new treatment technologies and combining low environmental impact and energy efficiency [7,8]. The design of sustainable wastewater treatment systems must focus on environmental protection, while minimizing energy and resource consumption [9]. Conventional wastewater treatment typically consists of a combination of physical, chemical, and biological processes and operations in order to remove solids, organic matter, and sometimes, nutrients from wastewater [10]. The physical processes include screening, sedimentation, and filtration, while the chemical processes include coagulation, flocculation, and disinfection. The biological processes involve the use of microorganisms to break down organic matter and nutrients in wastewater [11]. The combination of these processes and operations can effectively treat wastewater and reduce its potential impact on the environment and human health. However, conventional wastewater treatment plants cannot efficiently remove emerging pollutants such as drugs, hormones, and pesticides. Thus, many efforts have been made to develop effective technologies for wastewater treatment over the past few decades aimed at removing pollutants from wastewater and providing nontoxic but ecofriendly processes [12]. Different advanced wastewater treatment technologies, such as membrane filtration, adsorption, and advanced oxidation processes are being investigated to improve the removal efficiency of emerging pollutants and nutrients [13]. It is important to consider the advantages and disadvantages of different treatment technologies and their effectiveness in removing pollutants from wastewater when selecting a treatment process. Membrane technology has emerged as a favorite choice for reclaiming water from different wastewater streams for reuse [14]. The integration of resource recovery in wastewater treatment plants can also contribute to environmental sustainability by reducing waste and producing valuable resources [15].

## 2. Problems in Wastewater Treatment

WWTPs include different levels of treatment, starting with a primary treatment where part of the organic matter and suspended solids are removed, followed by a secondary treatment to eliminate biodegradable organic matter and nutrients, and, in some cases, ending with a tertiary treatment or advanced wastewater treatment to remove suspended solids and disinfect water [16]. However, many developing countries do not have complete wastewater treatment plants or only include primary (physical treatment) and secondary (biological treatment) stages without any tertiary treatment or advanced sludge processing [17]. In addition, inadequate WWTP design and operation can cause serious environmental problems both locally and globally [18].

Currently, the conventional activated sludge (CAS) processes are the most common treatments in WWTPs [19]. These treatments involve a large amount of energy due to the high electrical demand for aeration; on the other hand, the cost increases due to the necessary treatment of the resulting sludge [20,21]. In addition, in this aerobic treatment of activated sludge, the carbon content of the wastewater is not effectively utilized, resulting in its conversion into biomass and carbon dioxide without being fully exploited [22].

For energy and nutrient recovery from wastewater, anaerobic digestion is a promising treatment [23]. In such treatment, less sludge is generated, and less energy is consumed. In addition, anaerobic treatment is in line with the assumption of a circular economy, takes advantage of the organic matter content present in urban wastewater to produce biogas (i.e., a renewable energy source), and reduces CO_2_ emissions, compared to aerobic treatment [24].

However, despite the advantages referred to above, there are some difficulties in the application of anaerobic digestion for direct wastewater treatment. One of the difficulties is the low organic load of the wastewater, which causes a significant increase in digester heating per unit of biogas production and, therefore, directly influences the economic viability of the process [25,26,27,28]. Nevertheless, the limitations of anaerobic wastewater treatment can be overcome with processes that pre-concentrate the organic content and nutrients of the wastewater, thus turning cost-effective anaerobic treatment into biogas production and nutrient recovery [25,27,28,29,30,31].

This requires new developments and technologies to establish more energy efficient systems on water treatment and reuse, with membrane technology being a promising alternative [32,33].

## 3. Membrane Technologies

The development of synthetic membranes in the 1950s and 1960s led to the commercialization of membrane devices for industrial applications. Membrane technology has emerged as a favorite choice for reclaiming water from different wastewater streams for reuse [14]. The exact date when membrane technology was first used in wastewater treatment is not clear. Depending on the type of membrane, the selective separation of certain individual substances or substance mixtures is possible. In the simplest case, filtration is achieved when the pores of the membrane are smaller than the diameter of the undesired substance, such as harmful microorganisms.

The energy cost of membrane technology in water treatment varies depending on the type of membrane, the size of the plant, and the specific application [34]. While membrane technology can be energy-efficient compared to other treatment processes, it involves non-negligible capital and maintenance costs, and it needs intense redesign, that can altogether slow its adoption rate. The application of membrane technology for wastewater treatment and biofuel production not only reduces pollution but also decreases production costs. The cost–benefit analysis and technical efficiency evaluation of membrane bioreactor (MBR) technology for wastewater treatment showed that, with respect to the cost/energy efficiencies, the process is favorable [35]. Although, there are also some drawbacks, for example membrane fouling is a common issue in membrane technology, which can increase energy consumption and reduce the efficiency of the process [14]. Of course, it is important to consider in detail the energy cost and other factors when selecting a membrane technology for water treatment.

Different types of membranes, mainly using pressure as the driving force, are applied in water treatment processes: microfiltration (MF), ultrafiltration (UF), nanofiltration (NF), reverse osmosis (RO), and forward osmosis (FO) [14,36,37]. The main difference between these membranes is their pore size and the level of filtration accuracy they provide. Both FO and RO membranes are used for the separation of water from dissolved solutes, such as salts, and can be used in combination with other membrane processes, such as UF, NF or MF [38]. RO needs to be preceded by another of these processes, whereas FO can be used as a standalone process or as a step-in hybrid process as convenient. The selection of a membrane type depends on the specific application, the quality of the feedwater, and the desired level of filtration accuracy.

In FO processes, it is not necessary to apply external pressure since an osmotic pressure gradient is generated between the feed solution (FS) (for example, wastewater) and the draw solution (DS). This is an important advantage due to its lower energy consumption and due to the lower fouling of the membrane compared to pressure-driven processes [39,40,41]. In addition, the process has low fouling due to the nature of the driving force, and this slight fouling is mostly reversible [42]. However, if we take into account the energy consumption required to recover the DS to get rid of the salts to obtain clean water, the costs could approach those of RO. Thus, FO can concentrate wastewater and, consequently, organic matter and nutrients to feed subsequent anaerobic treatment to facilitate resource recovery.

This review focuses on the application of FO in wastewater treatment, especially on the elimination of contaminants present in urban wastewater, with the objective of contributing to the improvement of the management and optimization of water resources.

## 4. Forward Osmosis Development

### 4.1. Background of FO

**Figure 1:**
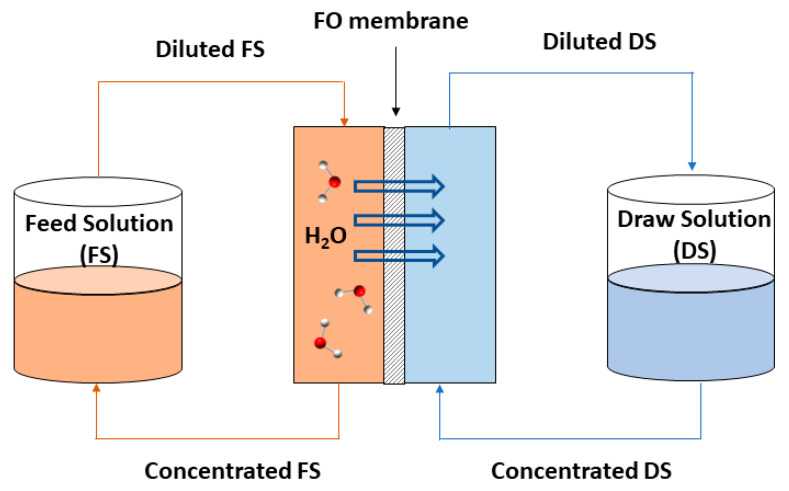
Scheme of the FO process.

FO, as an alternative membrane process in wastewater treatment, has attracted increasing interest in recent years. FO is the process in which water molecules pass through a semipermeable membrane, which separates two solutions, as shown in Figure 1. This transport and movement of molecules takes place due to the osmotic pressure difference (Δπ) which is the driving force in this phenomenon, as opposed to pressure-driven membrane processes. Thus, water is permeated passing through the membrane from the lowest solution concentration, FS, to the highest solute concentration solution, DS, while other solutes molecules are rejected [19,43]. FO has been investigated in various applications, such as seawater desalination [43], power generation [44], food processing [45], and wastewater treatment [46,47].

**Figure 2:**
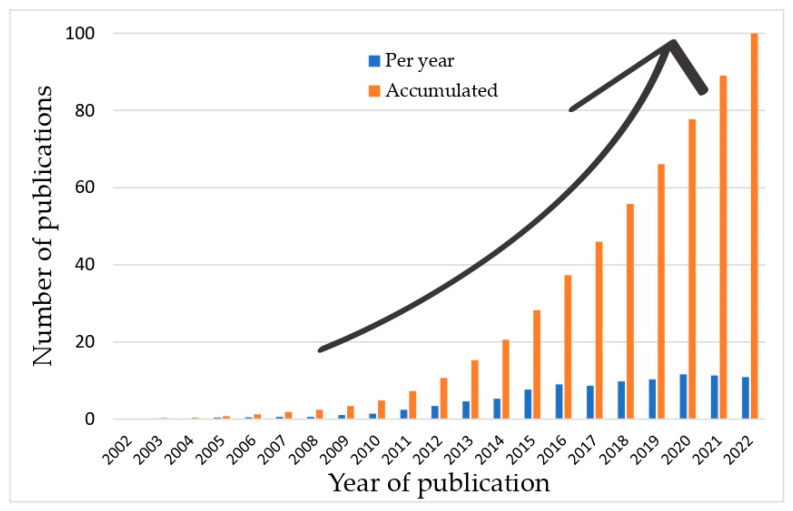
Yearly and accumulated numbers of publications on FO membranes (database: Scopus; search parameters: “forward osmosis membrane” in title, abstract, and keywords).

The beginning of the interest in FO dates back to the 18th century [48,49], while interest in this field has increased due to the commercialization of membranes designed for this process [2]. Figure 2 shows the rising interest in membranes of FO in the last 20 years by analyzing the number of publications on the topic.

### 4.2. Types of FO Membranes

Forward osmosis membranes are of interest if they have elevated water permeability while keeping salt retention high. In addition, they must present low concentration polarization, which is a phenomenon that, in the forward osmosis process, causes the osmotic pressure to decrease, leading to a reduction in the flow of water through the membrane. Furthermore, good chemical and mechanical stability to withstand working conditions is required [50].

FO membrane modules can be classified into plate and frame, spiral wound, tubular, hollow fiber, and flat sheet, according to the various geometric structures. The most used FO membrane modules are flat sheets and especially hollow fibers because these configurations require little space and are capable of separating large volumes, which are advantageous factors when compared with other membrane module configurations [51].

The most common FO commercial membranes, with respect to the material used, are cellulose acetate/triacetate (CA/CTA)-based membrane and thin-film composite (TFC) membranes of polyamide, polysulfone, or polyester layers [52,53,54,55,56,57]. A recent study proposed a classification of the emerging FO membranes into four categories according to their fabrication methods: cellulose acetate (CA), thin-film composites (TFCs), polybenzimidazole (PBI), and aquaporin (AQP), with TFCs the most competitive according to their properties [58].

The first commercialized FO membranes, i.e., CA/CTA membranes, have advantages such as good mechanical resistance, low tendency to fouling, good permeate fluxes, and high resistance to chlorine [59]. However, the operation pH range (3–8) is somewhat limited. To improve the characteristics of CA/CTA membranes, TFC membranes with a pH range of 2–11 and with higher permeate fluxes have been produced [2,51].

In addition to commercial membranes, numerous recent studies tried to modify the structure of the support layer using different methods or additives such as silica, graphene, zeolite, and TiO_2_ to improve the properties of commercial membranes [51,60,61,62,63].

### 4.3. Main Manufacturers of FO Modules

Various industrial companies offer FO membranes and commercial FO systems. Initially, the pioneering company for the supply of FO membranes was Hydration Technology Innovations (HTI) founded in 1986 in Albany (NY, USA). Later, another company called Oasys Water Inc. began to commercialize FO modules in the year 2010 in Cambridge (MA, USA). Another firm that manufactures FO membranes is FTS H2O™, also working in Albany (USA), specializing in CTA membranes in flat sheets. Next, the company Aquaporin Inside™ introduced FO membranes with aquaporin proteins that are highly selective, facilitating the transport of water molecules. These thin-film composite membranes are available in both flat sheet and hollow fiber configurations. In addition, Aquaporin A/S, a developer of these biomimetic membranes based in Lyngby (Denmark), recently signed a development agreement with another leading tubular membrane manufacturing company called Berghof Membrane Technology based in Leeuwarden (the Netherlands) to launch new membranes. Other companies have manufactured or have collaborated in the manufacture of FO modules such as Toray, Toyobo, Koch membrane systems, and Porifera, as well as some intermediary companies for marketing this type of module such as Sterlitech [64]. It should be noted that the supply of this type of FO membranes has facilitated studies and research related to FO that otherwise would have been much less developed today.

### 4.4. Important Factors

The operating conditions significantly affect the performance of FO. Therefore, their optimization is necessary to make the FO process more efficient. For example, it is necessary to optimize the concentration of DS and FS, the flow rates of FS and DS, the pH, the temperature, and the orientation of the membrane, which can be the active layer facing FS (AL-FS) or active layer facing DS (AL-DS). Furthermore, it is important to control the characteristics and properties of the membrane such as material, mechanical and chemical stability, active area, porosity, and hydrophobicity [65].

**Figure 3:**
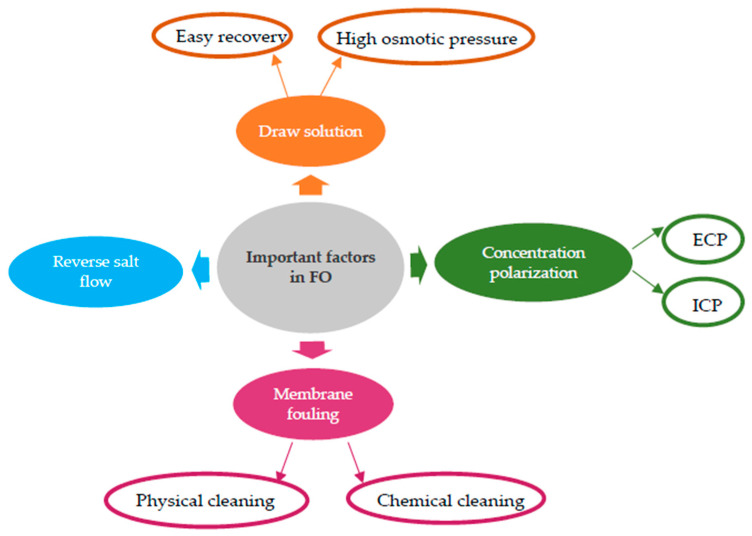
Important factors influencing the FO process.

In addition to the above, there are other relevant factors influencing the FO process that must be considered to solve possible drawbacks. Despite the wide variety of FO applications and the extensive FO-related research, there are some process issues and challenges that require still special attention for the process to maximize its commercial and industrial possibilities. These include the choice of the draw solution, the reduction in reverse salt flow, the regeneration of DS, and the reduction in concentration polarization and membrane fouling, as shown in Figure 3 [46].

#### 4.4.1. Draw Solution

To choose the possible draw solutions, it must be taken into account that they should meet a series of characteristics and requirements. Some important qualities are that it must generate high osmotic pressures [43,66], be economic, safe, and nontoxic, give minimal reverse draw solution flux, be stable, not react with the membrane material, and be easy to recover [67]. Commonly, solutes with a high solubility in water are selected to avoid their diffusion through the membrane. To improve the performance of the membrane by reducing concentration polarization on the surface of both sides, it is favorable to choose solutes with small molecular weight, giving low viscosity in the aqueous solution. Another important criterion, from an energetic point of view, is to have an easy and/or useful recovery or regeneration [67]. Extractive solutions with very varied solutes (inorganic salts, volatile compounds, organic solutes, etc.) have been suggested and studied. To date, most inorganic salt solutions as NaCl, MgCl_2_, KNO_3_, and MgSO_4_ have been tested due to their low cost and high osmotic pressure, with sodium chloride (NaCl) frequently selected as a reference DS for several reasons. First, it is generally used for standard membrane tests allowing a comparison of the results obtained with data from the literature because NaCl is commonly used as a DS. Furthermore, seawater and reverse osmosis concentrate are widely used as DSs in several interesting applications [68]. However, there are other interesting potential inorganic DSs depending on their characteristics and applications. For example, K_4_P_2_O_7_, KCl, and NH_4_PO_3_, which have the advantage of having fertilizing properties and providing high osmotic pressure, can be used as DSs if the end use of the water recovered is in irrigation. In this case, DS recovery would not be necessary [69,70], with subsequent economic savings. Organic-based solutes, compared to inorganic solutes, tend to have higher molecular weights, making their utilization somewhat more challenging. These solutes typically include sugars, diethyl ether, or organic salts. Studies have been conducted using common food additives such as monosodium glutamate (MSG), saccharin (SAS), and trisodium citrate (TSC), which generate slightly higher osmotic pressures but lower water flux than NaCl [71].

In addition, in some processes, gases such as CO_2_, SO_2_, and NH_3_ have been used due to their good solubility in water. However, they have not been implemented in real processes due to their limited osmotic pressure and high energy consumption requirements. There are also other less developed proposals for using magnetic solutes and hydrogels, which currently make the processes more expensive and are not sufficiently understood [72,73].

At present, the choice of DS and its regeneration are key issues in the application of FO. Energy-consuming solute recovery is one of the major considerations in selecting the DS. Some regeneration methods may consist of their direct use without ulterior recovery [74]. In some cases, DS is regenerated by membrane separation, such as RO [75], NF [76], UF [77], MD [78], ED [79], chemical precipitation [80], or thermal separation [81]. Other options are magnetic recovery and electrolytic recovery. Although there are various methods for DS regeneration, each method has its advantages and limitations for the application of the FO process [51].

#### 4.4.2. Reverse Salt Flow or Reverse Solute Diffusion

Another important requirement to be taken into account in the selection of the extraction solution is to minimize the diffusive transport of salt; this is to reduce, as much as possible, the flow of salt from the DS to the FS [82]. To experimentally calculate the reverse salt flow $J_{S}$, Equation (1) is usually used [8].

(1) $$J_{S}=\frac{C_{FSt_{i+1}}V_{FSt_{i+1}}-V_{FSt_{i}}V_{FSt_{i}}}{A\left(t_{i+1}-t_{i}\right)},$$

where $C_{FSt_{i+1}}$ is the salt concentration of the feed solution at time $t_{i+1}$, $C_{FSt_{i}}$ is the salt concentration of the feed at time $t_{i}$, $V_{FSt_{i+1}}$ and $V_{FSt_{i}}$ are the feed volumes at times $t_{i+1}$ and $t_{i}$, respectively, and the surface area of the active side of the membrane is A.

This flow is known as reverse solute diffusion (RSD), where the solute passing through the membrane from the DS to FS causes a decrease in the driving force for water flow and increases membrane fouling via a cake-like mechanism [83].

RSD is unavoidable in the FO process but should ideally be minimal [84]. This is affected by the DS physicochemical properties (for example, ion/molecule size, viscosity, ion charge, and diffusivity) [85], flow rate [86], membrane structure characteristics (e.g., thickness and porosity) [87], concentration polarization [88], etc. Eventually, RSD would alter the chemistry and composition of the FS [42]. For example, the flow of chlorides when NaCl is used as a DS with real urban wastewater as the FS hinders the correct determination of organic matter and interferes with or even inhibits subsequent anaerobic treatment [89,90].

Although it is impossible to eliminate RSD, it can be reduced and mitigated by choosing a less permeable extraction solute, developing specific advanced membranes, or optimizing the operation conditions [83]. However, many studies lack RSD data, making it difficult to explore and understand how to mitigate solute reverse flow.

#### 4.4.3. Concentration Polarization

Concentration polarization is an inevitable and common phenomenon in both osmosis processes and pressure-driven membrane processes [46,91,92]. This phenomenon, in osmotic processes, takes place due to the difference in concentration between the feed solution and the extraction solution that separates an FO membrane.

Concentration polarization (CP) can occur in two ways in FO processes: external concentration polarization (ECP) and internal concentration polarization (ICP). Commonly, ECP occurs on the surface of the active layer of the membrane, and ICP occurs within the porous support layer of the membrane. Furthermore, there are two types, concentrative CP and dilutive CP, depending on the orientation of the membrane. For FO in AL-FS mode, concentrative ECP and dilutive ICP take place, whereas, in AL-DS, dilutive ECP and concentrative ICP take place. In pressure-driven membrane processes, the difference is that only concentrative ECP can take place [46,51]. However, regardless of membrane orientation, both ICP and ECP occur simultaneously. CP, in the FO process, influences water flow, salt reverse flow, and contaminant retention.

In FO, the water flux in AL-FS mode (when the FS is in contact with the active layer and the DS is in contact with the support stratum) can be calculated using Equation (2) [43,93].

(2) $$J_{w}=A\left[{\;\pi }_{\mathrm{Draw},b}\exp\left(-J_{w}K\right)-{\;\pi }_{\mathrm{Feed},b}\;\exp\left(\frac{J_{w}}{k}\right)\right].$$

The water flux in AL-DS mode can be expressed as Equation (3).

(3) $$J_{w}=A\left[{\;\pi }_{\mathrm{Draw},b}\exp\left(-\frac{J_{w}}{k}\right)-{\;\pi }_{\mathrm{Feed},b}\;\exp\left(J_{w}K\right)\right],$$

where $J_{w}$ is the water flux, A is the pure water permeability coefficient, $\pi_{\mathrm{Feed},b}$, $\pi_{\mathrm{Draw},b}$ are the osmotic pressure of FS and DS in the bulk solution, K is the solute resistivity for diffusion within the porous support layer, and k is the mass transfer coefficient.

In the FO process, the appearance of ECP, which usually occurs on the surface of the active layer, can decrease the transmembrane osmotic pressure difference, resulting in decreased water flux. Optimizing the flow of water and improving parameters such as flow velocity or turbulence could reduce or mitigate ECP [46,94].

The ICP that takes place in the support layer is associated with porosity, hydrophobicity, membrane thickness, tortuosity, and other membrane characteristics [95]. Therefore, the characteristics of the membrane must be considered, since they can increase consumption and operating costs by requiring exhaustive cleaning due to fouling [2,51]. In fact, these possible drawbacks are comparatively of low impact because FO is characterized by low fouling and high energy efficiency.

#### 4.4.4. Membrane Fouling

Membrane fouling is unavoidable for most membrane processes [51,96], but it is key when membranes are used for the treatment and desalination of wastewater. Regarding membrane fouling, FO has emerged as one of the promising membrane processes and alternatives to reverse osmosis (RO). It should be noted that the formation of a cake layer on the membrane surface is common in FO and RO processes. However, in the case of RO, the cake layer must be compacted under pressure, making it more irreversible compared to FO. The non-compaction nature of FO allows tangential flow across the membrane surface to combat fouling more effectively [97].

Factors such as membrane orientation, hydrophobicity, charge, material, feed substrate, and operating conditions or flow direction can influence membrane fouling [98,99]. There are different types of membrane foulants in the feed solution, such as colloidal or particulate matter, inorganic or organic components, chemical reagents, microorganisms, and microbial species, with colloidal fouling being the predominant fouling mechanism in urban wastewater treatment [97].

Fouling is an important factor in FO, since it reduces the flow of water and the efficiency and useful life of the membrane. The fouling of the membrane, in addition to affecting the reduction of the water flow, also affects the retention of contaminants present in the feed solution, for example, when using municipal wastewater as a feed solution [8]. This is because it can improve the retention of contaminants that remain retained or adsorbed on the active surface of the membrane, due to chemical interactions that take place between the contaminant and the membrane [51,95,100].

In this sense, there has been a recent increase in publications related to the fabrication and modification of membranes to minimize fouling, to increase the flow of water without increasing the reverse flow of salt, i.e., to improve the properties of commercial membranes with antifouling or antibacterial characteristics. Some studies showed the incorporation of functionalized hydrophilic nanomaterials into the membrane [101] or surface coating [102] to be effective methods to improve the performances of membranes [64,97]. For example, many nanomaterials (such as zeolite [63], metal or metal oxide nanoparticles [103], or graphene oxide [104]) have been used to fabricate membranes, enhancing both the permeability and the antifouling capacity [105].

In addition, to remediate the consequences that fouling of the membrane could have on the water flow, membrane cleaning methods are necessary to recover the water flow [94]. Cleaning methods can be physical cleaning, chemical cleaning, or a combination of both [51]. Physical cleaning can consist of surface washing or/and osmotic backwashing. Fouling is generally reversible, and the initial flow can be recovered by physical cleaning at high flow rates after short-term experiments [106]. Physical cleaning has great advantages when the fouling is superficial; however, if the fouling is strongly adhered to the membrane, physical cleaning is ineffective, and chemical cleaning is necessary [42]. Chemical cleaning requires the use of commonly used chemical reagents such as NaOH, HNO_3_, and NaOCl. However, the use of reagents may decrease membrane life due to modifications in the membrane material, as well as facilitate subsequent irreversible fouling, or it may not completely eliminate membrane fouling [51].

Although, to mitigate fouling, there are possibilities such as optimization of process parameters and cleaning methods, or membrane modifications to improve antifouling properties, as discussed above, there is another way to avoid fouling: feed solution pretreatment [8,107,108].

In summary, it can be concluded that the fouling and cleaning of the membrane are among the drawbacks of FO since it increases the cost and energy consumption of the operation [109]. Although additional studies and research are required to understand the fouling mechanism in forward osmosis during long periods of operation on an industrial scale [64], recent studies have shown that, with new materials, the fouling of FO membranes is a reversible process in many cases [110,111].

## 5. Wastewater Contamination

Pollution is one of the most important environmental problems that affect our world, and it is the result of the introduction of substances into the environment in such a quantity as to cause adverse effects in humans, animals, plants, or materials exposed to doses that exceed acceptable levels in nature [34].

Traditionally the environment has been divided, for its study and interpretation, into three components: air, water, and soil. However, this division is merely theoretical since most pollutants interact with more than one element in the environment [112]. The sources of contamination can be natural sources or of anthropogenic origin such as industrial, commercial, agricultural, and domestic activities.

### 5.1. Contaminants of Emerging Concern or Micropollutants

The interest in contaminants of emerging concern (CECs) has grown in recent decades. They are organic pollutants that are present in the environment in increasing concentrations and can cause damage to the environment and human health [113,114].

Contaminants of emerging concern are not necessarily new chemicals and generally include contaminants that have been present in the environment, but whose presence, significance, and effects (toxicity) are only now being evaluated. Previously, some of these compounds were not included in environmental legislation because they were previously not easily detected due to the lack of sufficiently robust analytic methods. However, thanks to new methodologies and increasing knowledge on their effects, concentration limits are beginning to be considered and established; therefore, some of these pollutants have now been included in environmental legislation [115,116,117,118].

The main sources of emerging pollutants are of anthropogenic nature. They are derivatives of agriculture such as pesticides and veterinary drugs or compounds found in cattle food additives. There are also pharmaceutical and personal care products (PPCP) that the population uses daily. Discharges of effluents from hospitals, industrial plants, and urban WWTPs are highly relevant to the aquatic environment due to the presence of this type of contaminant in their effluents [119,120,121,122]. All these sources can cause occasional contamination, but pollution can also spread by seeping into surface and groundwater from rainfall, soil infiltration, and surface runoff.

Although WWTPs are designed to remove solid materials and to reduce levels of metals, bacteria, and other pathogens, most are not designed to specifically remove organic contaminants. Numerous studies around the world have detected the presence of different groups of pollutants in wastewater, and significant concentrations of pollutants are detected in both influents and effluents in concentrations from the ng/L to the mg/L range [114,123,124,125,126,127]. The concentration of each pollutant varies from one plant to another depending on the country, the size of the plant, the population, and many other factors.

The list of pollutants of emerging interest or micropollutants includes a wide variety of compounds with different structures and uses, as well as metabolites and transformation products. Table 1 shows the most representative contaminants of emerging interest that have been found in WWTPs [128].

#### 5.1.1. Environmental Effects

It is known that the presence of CEC in the aquatic environment potentially affects aquatic organisms and can cause changes that threaten the sustainability of aquatic ecosystems [113,129]. The presence of pollutants in the environment can cause negative biological toxic effects on organisms such as mutagenicity, estrogenicity, and genotoxicity. Many of them are toxic or are classified as endocrine disruptors, which implies that exposure to them can lead to alterations in the growth, development, reproduction, and behavior of living organisms [130].

Some of these effects cause the inhibition of the growth rate of the organism or the masculinization of marine gastropods, producing a decrease in the population. For example, carbamazepine can alter metabolic activities, slow growth, reduce fecundity, and alter steroid levels in fish [131]. Exposure to diclofenac in fish may adversely affect cardiovascular development and cause oxidative stress or a reduction in steroid hormones. UV filter compounds cause endocrine-disrupting effects as they are capable of interfering with the thyroid axis and the development of reproductive organs, as well as the brain, in both aquatic and terrestrial organisms [132].

**Figure 4:**
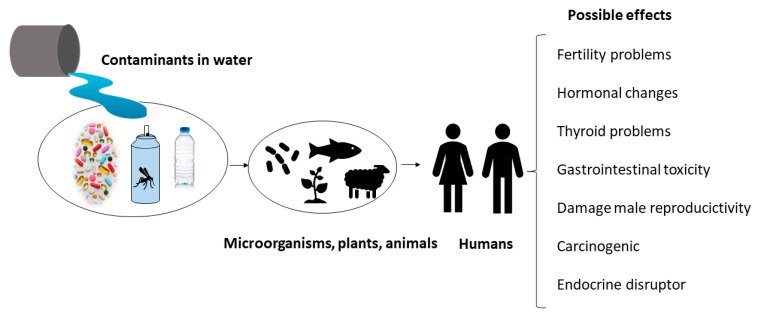
Some harmful effects on human health of emerging contaminants.

Figure 4 shows some of the possible effects that contaminants cause in humans because of the food chain.

#### 5.1.2. Ecotoxicological Risk Evaluation

In general, environmental risk depends on three factors [133]:

- Amount of the substance present in the environment (for example, soil, water, or air).
- Exposure time of the receptor with the contaminated environment.
- The inherent toxicity of the substance.

In the evaluation of environmental risk, data and observations are collected on the harmful effects that toxic substances can generate toward the environment and health, in order to be able to assess the risk they imply. The evaluation consists of obtaining data to determine the dose of exposure of an organism to a contaminant and the response that this will cause. Empirical dose–response data are compared with the exposure received by humans or other living organisms, to have a complete evaluation of the risk generated in a certain contaminated environment [134,135].

**Figure 5:**
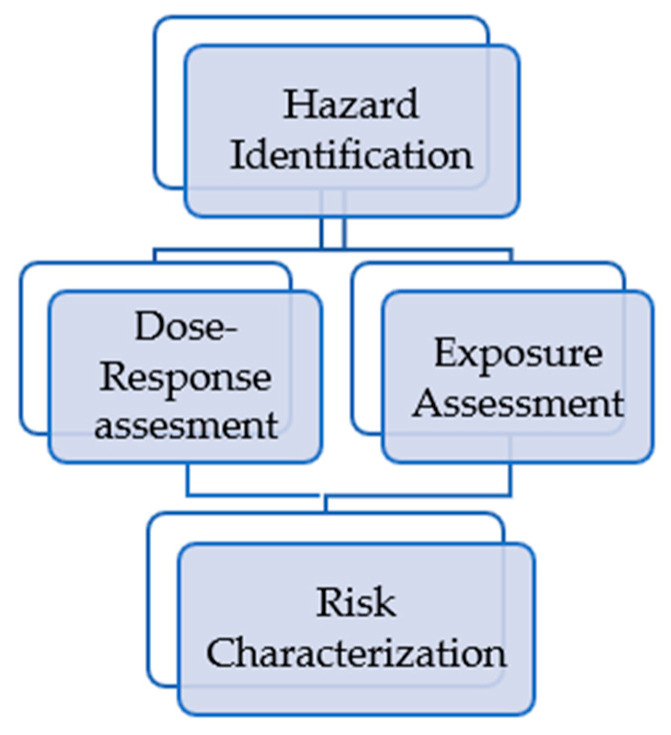
Stages of environmental risk assessment.

Environmental risk assessment can be as suggested by the EPA (Environmental Protection Agency) guide [134,135,136], which divides the process into four steps (Figure 5).

For dose–response analysis, the following toxicology concepts are used:

– Median effective concentration (EC_50_): Concentration obtained statistically or graphically estimated that causes a given effect in 50% of the group of organisms, under specified conditions.
– Median lethal concentration (LC_50_): Statistically derived or graphically estimated concentration (in air or water) that causes death, during exposure or within a defined period after exposure, of 50% of the group of organisms during a given period and other specific conditions. LC_50_ is generally expressed in mg/L.
– Median lethal dose (LD_50_): Individual dose of a substance that is statistically or graphically estimated to be lethal to 50% of the group of organisms under specified conditions. Generally, LD_50_ is expressed in mg/kg of body weight.
– No observable effect level (NOEL): The highest concentration or amount of a substance found experimentally or by observation that does not cause alterations in the morphology, functional capacity, growth, development, or life span of organisms, distinguishable from those observed in organisms normal (control) samples of the same species and strain, under conditions identical to those of exposure.

Given the great complexity of aquatic ecosystems, it is not possible to assess the effect of pollutants on all the organisms that live in them. For this reason, in order to assess the individual effects of pollutants, test species representative of the ecosystems are used. The choice of the test species is made considering a series of criteria, such as ecological importance, sensitivity to contaminants, or feasibility of growing in laboratory conditions. One of the most widely used organisms to perform toxicity bioassays is the genus Daphnia. This organism plays an important role in the trophic chain of freshwater systems, being the dominant consumer of primary producers, and it is an important source of food for vertebrate and invertebrate predators [137].

Generally, three aquatic organisms are studied (fish, green algae, and Daphnia magna) as standard species in recommended by the EC (European Commission), OECD (Organization for Economic Cooperation and Development), and ISO (International Organization for Standardization) ecotoxicity tests. In addition, they are presented as bioindicators to assess environmental risk, since they belong to three different orders of the food chain, giving an idea on how the concentration of contaminants affects the different levels of the aquatic food chain [138].

For the ecological risk assessment, an estimated risk ratio (*RQ*) can be calculated for each CEC using Equation (4).

(4) $$Risk\;Quotient\;\left(RQ\right)=\frac{C_{X\;\left(EFFLUENT\right)}}{PNEC}.$$

The variable *C_X_* (effluent) represents the concentrations in the final treated effluent (in ng·L^−1^), and *PNEC* represents the predicted no-effect concentrations (in ng·L^−1^), which until now were not always available in the literature. Thus, *PNEC*s are calculated on the basis of toxicity data, such as LC_50_ or EC_50_, and the safety factor (AF), which is typically 1000 for short-term toxicity data, as recommended by the Water Framework Directive [139,140]. If the *RQ* is <0.1, it indicates low risk; if the *RQ* is between 0.1 and 1.0, it corresponds to moderate risk; if the *RQ* is ≥1.0, it indicates high risk [141,142].

### 5.2. Options to Contaminants of Emerging Concern Removal in Wastewater

Conventional WWTPs have been designed to eliminate eutrophic contamination to avoid excessive organic and mineral nutrients that could support an overabundant plant life, which in the process of decaying would deplete the oxygen supply. However, they are not designed to eliminate these new micropollutants; hence, additional treatments are required for their elimination before their introduction to surface waters [131,143,144,145]. Thus, additional techniques to remove the emerging contaminants need to be implemented.

In addition, we must keep in mind and be aware that CECs have a wide variety of chemical properties; therefore, removal success varies depending on their particular properties. Wastewater treatment is a more complicated process than water treatment due to the characteristics of wastewater that must be thoroughly considered so that it can be safely integrated into the environment [146].

**Figure 6:**
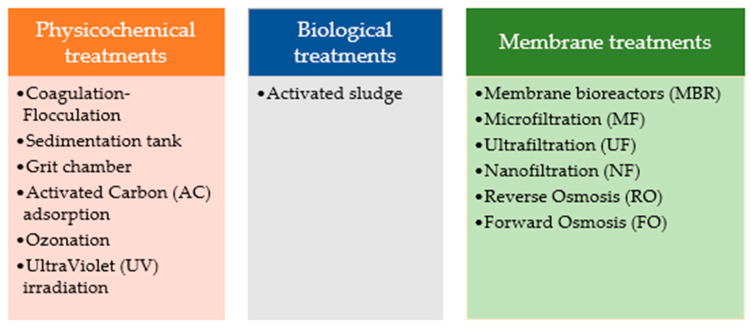
Treatments to remove contaminants from the wastewater.

There are different urban wastewater treatments for the removal of pollutants as shown in Figure 6.

– Physicochemical treatments: The coagulation–flocculation process has been found to be unable to remove contaminants, in addition to existing techniques in WWTPs such as grit chambers or sedimentation tanks to remove solid particles, ash, and other suspended solids [147]. This group of physiochemical treatments also includes processes such as activated carbon (AC) adsorption or ultraviolet (UV) irradiation. Another example in this field involves advanced oxidation technologies that can eliminate some of these microcontaminants from residual waters such as ozonation. Although oxidation is a promising process for removing pollutants from wastewater, especially using chlorine or ozone, the reaction of these chemicals produces byproducts, and the effects of these byproducts are unknown. Therefore, special care must be taken when using these chemicals for wastewater treatment [148].
– Biological treatments: Activated sludge can convert organic compounds into biomass, among other compounds. However, while this is a great achievement, not all compounds are completely broken down into biomass in this process. Biological treatment is a common method for wastewater treatment that uses microorganisms to remove pollutants. However, it is only capable of removing a part of a wide range of emerging pollutants [149].
– Membrane treatments: These include membrane bioreactors (MBR) and membrane filtration processes [150,151]. Pressure-driven membrane techniques such as microfiltration (MF), ultrafiltration (UF), reverse osmosis (RO), and nanofiltration (NF) have also been used to treat water contaminated with micropollutants [152]. Both NF and RO can remove contaminants such as suspended and dissolved solids, organic matter, viruses, and bacteria, but RO is additionally capable of eliminating smaller molecules such as ions. However, these processes, due to membrane concentration polarization and the high hydraulic pressures required, have high costs and are difficult to scale [92]. A possible alternative to overcome the disadvantages of pressure-driven membrane techniques could be the use of FO processes [153]. In the forward osmosis process, the driving force is the osmotic gradient rather than the pressure-driven force, which could be an important advantage with respect to membrane fouling, as already mentioned. In this process, the osmotic pressure gradient facilitates the passage of water across a semipermeable membrane between a concentrated extraction solution and a less concentrated feed solution, while retaining other solutes. This leads to dilution of the extraction solution, while the solutes in the feed stream become concentrated [43,154].

#### Forward Osmosis in the Removal of Contaminants of Emerging Concern from Wastewater

FO has shown promising potential in the removal of various contaminants from water sources. As previously commented, the absence of applied hydraulic pressure could reduce operational and energy costs and provide a better fouling control than high-pressure-driven membrane processes due to physically reversible fouling. There are several recent studies that corroborated the feasibility of this membrane process in the elimination of contaminants in water [8,93,100,154,155,156,157,158,159,160,161,162,163,164,165,166,167,168,169,170,171,172,173,174,175]. In these studies, membranes of different configuration, different materials, and different contaminants were used, all of which had good contaminant removal in common. For example, a study by Cartinella et al. (2006) focused on the removal of two hormones (estrone and estradiol) using a CTA flat sheet FO membrane. The results demonstrated hormone rejection between 96% and 97% [157]. Another study by Salamanca et al. (2021) using TFC hollow fiber FO membranes with aquaporin inside focused the rejection of 24 contaminants. The study demonstrated remarkable rejection rates, exceeding 93% for all the tested compounds [93].

In previous studies, most investigations regarding FO for contaminant removal have predominantly focused on clean or synthetic water samples. However, there are few examples in the literature that examined the application of FO using real urban wastewater as the feed solution. Nonetheless, a limited number of studies have explored this aspect. To provide an overview of these investigations, we present Table 2 summarizing the relevant studies in which real wastewater was employed as the feed solution in FO processes, categorized according to the year of publication. This table serves as a valuable resource in understanding the practical applications of and challenges associated with using FO in real-world wastewater scenarios.

Table 2 displays recent studies conducted between 2011 and 2023, focusing on FO applications in real wastewater. It can be found that NaCl or synthetic seawater are commonly used as DSs, while membrane materials such as CTA and TFC membranes are frequently employed. The location of the feed solution indicates the countries where the studies were conducted, including Australia, Chile, China, Japan, the Netherlands, Spain, Sweden, and the United Kingdom. It is worth mentioning that, among the investigations using real wastewater as a feed solution, only a limited number examined the contaminants present in the water and their removal efficiency [8,176,184,187], as shown by a √ sign in Table 2 in the column of contaminants.

**Figure 7:**
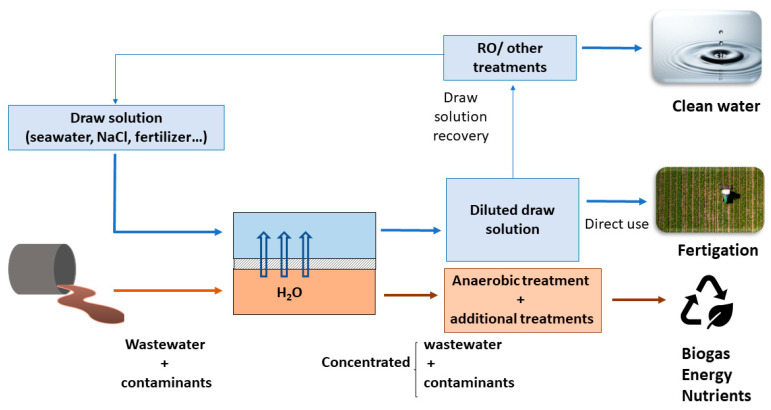
Diagram of the integration of FO as a process of concentration in the wastewater treatment to obtain resources and reuse of water.

In utilizing FO for the treatment of urban wastewater, an opportunity arises to obtain a concentrated solution containing organic matter and contaminants. This concentrated solution can then be directed toward additional processing, such as anaerobic treatment. By doing so, valuable resources can be extracted from the organic matter, leading to the generation of biogas. Additionally, some contaminants present in the concentrated solution can be effectively removed and degraded, further enhancing the overall treatment efficiency and environmental benefits [190]. However, the appearance of emerging contaminants in sludge can eventually inhibit anaerobic digestion and can induce health problems when sludge is recycled to agriculture, requiring methods to remove contaminants either before or after anaerobic treatment. Some of the pollutant remediation methods include electrooxidation, ultrasonication, thermal hydrolysis, ozonation, and bioaugmentation [191]. Concurrently, a diluted DS would be obtained, which, depending on its composition and intended application, can be regenerated, subjected to desalination processes to yield clean or potable water, or even utilized directly as fertilizer for irrigation purposes. This holistic approach presents a pathway toward resource recovery and the sustainable management of wastewater as shown in Figure 7.

Hence, it is crucial to promote the practical implementation of FO technology for water and wastewater treatment. This entails exploring a wider range of DS and conducting studies on contaminants present in real wastewater. By expanding the scope of research in these areas, the potential industrial applications of FO can be further extended. Moreover, this is a critical step toward the promotion of commercial markets for the FO process, unlocking its full potential and addressing the diverse needs of water treatment in various sectors.

## 6. Concluding Remarks and Future Perspectives

In conclusion, this systematic review provided valuable insights into the significant capability of FO as a promising technology in water and wastewater treatment such as contaminant removal, resource recovery, energy efficiency, and integration potential. The review highlighted the crucial role played by FO in removing contaminants from wastewater, thus enabling the production of clean water without the risk of contamination. The advantages of FO, including its low energy consumption, reversible low fouling, and high contaminant rejection rates, place it as an attractive alternative to conventional wastewater treatment methods. These advantages highlight the potential of FO to contribute to sustainable water management and address the challenges associated with water scarcity and pollution. In the future, further research and development in the field of FO to optimize the systems and overcome existing challenges would help to improve the technology and expand its practical applications. Some key areas for future exploration and improvement are the following:

– Focusing on optimizing FO systems by developing advanced membrane materials, exploring innovative fouling mitigation strategies, and investigating novel approaches for the recovery of draw solutions, as well as working with real wastewater to work in real conditions.
– Process integration and hybrid systems by exploring the integration of FO with other water treatment processes, e.g., reverse osmosis or electrochemical processes, to improve overall treatment efficiency. Hybrid systems that combine FO with other technologies may offer unique advantages for specific applications.
– Scaling up and commercialization by advancing FO from laboratory-scale to large-scale implementation, which will require addressing engineering challenges and optimizing system designs.

Collaborative efforts between academia and industry will be essential to drive progress in FO research and promote its implementation on a larger scale. With further research and innovation, FO can find practical applications in desalination, industrial wastewater treatment, resource recovery, and water reuse.

## Figures and Tables

**Table 1:** List of major emerging contaminant groups found in municipal wastewater.

Emerging Contaminant Group	Examples
Antibiotics	Ciprofloxacin, ofloxacin, sulfamethoxazole, sulfadiazine, metronidazole, erythromycin, clarithromycin, amoxicilin
Analgesics/ antiinflammatories	Diclofenac, naproxen, ibuprofen, salicylic acid, acetaminophen
Lipid regulators	Clofibric acid, gemfibrozil
Psychiatric drug/anticonvulsants	Carbamazepine
Antimicrobials	Triclosan
Hormones	17-α-Etinilestradiol (EE2), 17-β-estradiol (E2), estrone (E1), progesterone
X-ray contrast	Iohexol, iopromide
Stimulants	Caffeine
Anti-itching	Crotamiton
Insect repellant	DEET (N,N-diethyl-meta-toluamide)
Herbicides	Atrazine
Artificial sweeteners	Acesulsame, sucralose, aspartame
Preservatives	Methylparaben, ethylparaben
Cardiovascular drug	Propranolol
Plastics additives	Bisphenol A
Surfactants	4-tert-Octylphenol, 4-nonylphenol
UV filters	Benzophenone

**Table 2:** Publications per year when feed solution in FO process is from WWTP.

Year	Membrane Configuration	Membrane Material	Supplier	FS Location	DS	Contaminants	Reference
2011	Spiral wound	CTA with embedded polyester mesh	HTI	WWTP Amsterdam West (The Netherlands)	NaCl and MgCl_2_·6H_2_O	-	[106]
2013	Flat sheet	CTA with embedded woven mesh	HTI	WWTP Wollongong (New South Wales, Australia)	NaCl	√	[176]
2014	Spiral wound	CTA with embedded polyester mesh	HTI	WWTP Queensland (Australia)	NaCl	-	[177]
2015	Three flat-sheet membranes (TFC, CTA-1, and CTA-2)	TFC with polyamide on polysulfone with embedded support CTA-1 with embedded polyester mesh CTA-2 with embedded nonwoven support	HTI	WWTP (Japan)	Synthetic seawater	-	[178]
2016	Three flat sheet membranes	CTA with embedded polyester mesh CTA with embedded nonwoven mesh TFC with embedded polyester screen support	HTI	WWTP Temuco (Chile)	NaCl	-	[179]
2016	Spiral-wound	CTA	HTI	WWTP Shanghai (China)	NaCl	-	[180]
2016	Flat sheet	CTA with embedded polyester mesh	HTI	WWTP (Japan)	Synthetic seawater	-	[181]
2016	Flat sheet	CTA with embedded polyester mesh	HTI	WWTP (China)	Synthetic seawater	-	[182]
2018	Flat sheet	TFC with polysulfone with embedded support	Porifera	WWTP New South Wales (Australia)	NaCl and NaOAc	-	[25]
2018	Flat sheet (proprietary, PFO-100)	ABS (wetted), carbon fiber (structural) with aquaporins	Porifera	WWTP (Sweden)	NaCl	-	[183]
2018	Flat sheet	CTA with embedded polyester mesh	HTI	WWTP, Beijing (China)	NaCl	√	[184]
2018	Flat sheet	CTA	HTI	WWTP (China)	Synthetic seawater	-	[185]
2019	Spiral wound	TFC	Toray	WWTP Valencia (Spain)	NaCl and MgCl_3_	-	[186]
2019	Flat sheet	CTA with embedded polyester mesh	HTI	WWTP Beijing (China)	NaCl	-	[89]
2019	Flaat sheet	TFC	Homemade	WWTP Jinan (China)	Synthetic seawater	-	[107]
2021	Spiral wound	TFC	Toray	WWTP Girona (Spain)	Sea salt	√	[187]
2022	Hollow fiber and flat sheet	TFC	Singapore Membrane Technology Centre	WWTP Southampton (UK)	NaCl	-	[188]
2022	Hollow fiber	TFC with aquaporins	Aquaporin A/S	WWTP Valladolid (Spain)	NaCl, MgSO_4_·7H_2_O, C_6_H_12_O_6_, CH_3_COONa, and MgCl_2_·6H_2_O	√	[8]
2022	Flat sheet	CTA with embedded polyester mesh	HTI	WWTP Temuco (Chile)	NaCl	-	[189]
2023	Tubular (TFO-D90)	PVC with aquporins	Berghof Membrane Technology GmbH	WWTP Valladolid (Spain)	NaCl	-	[90]

## Data Availability

Not applicable.
